# Solvation Mediates the Mechanisms of Homogeneous Co-Electrocatalysis at Interfaces

**DOI:** 10.1021/acs.jpclett.6c02610

**Published:** 2026-09-30

**Authors:** Megan E. Moberg, Robert Sacci, Benjamin Doughty, Charles W. Machan

**Affiliations:** † Department of Chemistry, 2358 University of Virginia, PO Box 400319, Charlottesville, Virginia 22904-4319, United States; ‡ Chemical Sciences Division, 6146 Oak Ridge National Laboratory, Oak Ridge, Tennessee 37831, United States

## Abstract

Electrochemical reactions occur near electrode surfaces—this interfacial environment has long obscured the mechanistic insight required to improve selectivity and overall rates. While traditional linear spectroscopies can be used to probe bulk chemical changes, the interfacial response is buried within, limiting insight into solvation environments that modulate homogeneous electrochemical transformations. Here, we address these challenges by studying the protic catalytic mechanism of a Cr-based bipyridine catalyst capable of the selective reduction of carbon dioxide (CO_2_) to carbon monoxide (CO), at an applied potential using sum frequency generation (SFG) vibrational spectroscopy. Measurements on a series of increasingly complex samples allowed for the characterization of species and solvent environments in an electrochemical solution at a gold working electrode surface. SFG spectra show how applied potential affects the packing of molecules and their conformations at interfaces to allow catalytic species to approach and transfer electrons—emphasizing the divide between species in the bulk versus at the surface and underscoringthe need for further research in this area.

## Graphical abstract

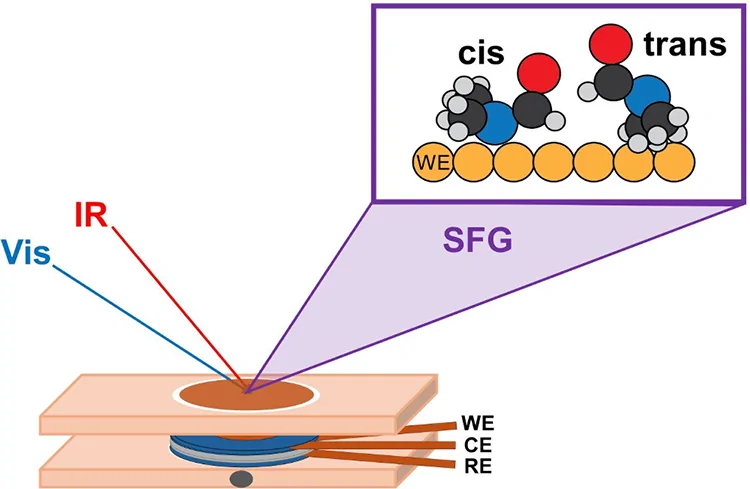

The amount of accumulated atmospheric carbon dioxide (CO_2_), a leading contributor to the global climate crisis, has grown to 1.5 trillion tons^1^ since the start of the industrial revolution. This crisis can potentially be mitigated by electrochemically reducing CO_2_ to useful, value-added chemical feedstocks such as carbon monoxide (CO) and carbon-based precursors.^2^ Initial attempts to develop molecular electrocatalysis for the CO_2_ reduction reaction (CO_2_RR) utilized late transition metals like Pd,^3^ Ru,^4^ and Re;^5^ however, due to their scarcity and cost, recent focus has switched to more earth-abundant transition metals like Mn,^6,7^ Fe,^8,9^ Co,^10^ and Ni.^11^ While the Cr/Mo/W triad is underrepresented in the field, the Machan group has published multiple Cr-based catalysts for the selective reduction of CO_2_ to CO.^12–19^ The first Cr-based catalyst was Cr­(^*tbu*^dhbpy)­Cl­(H_2_O) (**1**), where the ligand precursor (^*tbu*^dhbpy­(H)_2_) is 6,6′-di­(3,5-di-*tert*-butyl-2-hydroxybenzene)-2,2′-bipyridine.^12^ The catalytic cycle, proposed by Hooe *et al*., proceeds under protic conditions (Figure 1) as follows:^12,13^ first, the Cr complex undergoes a stepwise two-electron reduction with chloride loss forming a four-coordinate monoanionic Cr­(II)­(bpy^•–^) active species (*i*), which CO_2_ can bind forming [Cr-CO_2_]^−^ (*ii*). Next, the CO_2_ adduct is protonated to form a hydroxycarbonyl species, [Cr-CO_2_H] (*iii*). Upon reduction of *iii* to [Cr-CO_2_H]^−^ (*iv*), a second proton transfer is initiated: rate-determining C–OH bond cleavage to generate a carbonyl complex (*v*) with H_2_O as a co-product (the CO_2_ binding step also shares a portion of rate control). The release of CO is facile and regenerates the neutral four-coordinate species (*iv*) to close the catalytic cycle. Under optimized catalytic conditions, preparative experiments have assessed that **1** can selectively produce CO from CO_2_ with a turnover frequency (TOF) of 7.12 s^–1^.^20^ More recently, other Cr catalyst platforms have been reported which are based on quaterpyridine, with the complexes formed with the neutral ligand framework exhibiting lower stability in the presence of the accumulating CO product.^21,22^

**Figure 1:**
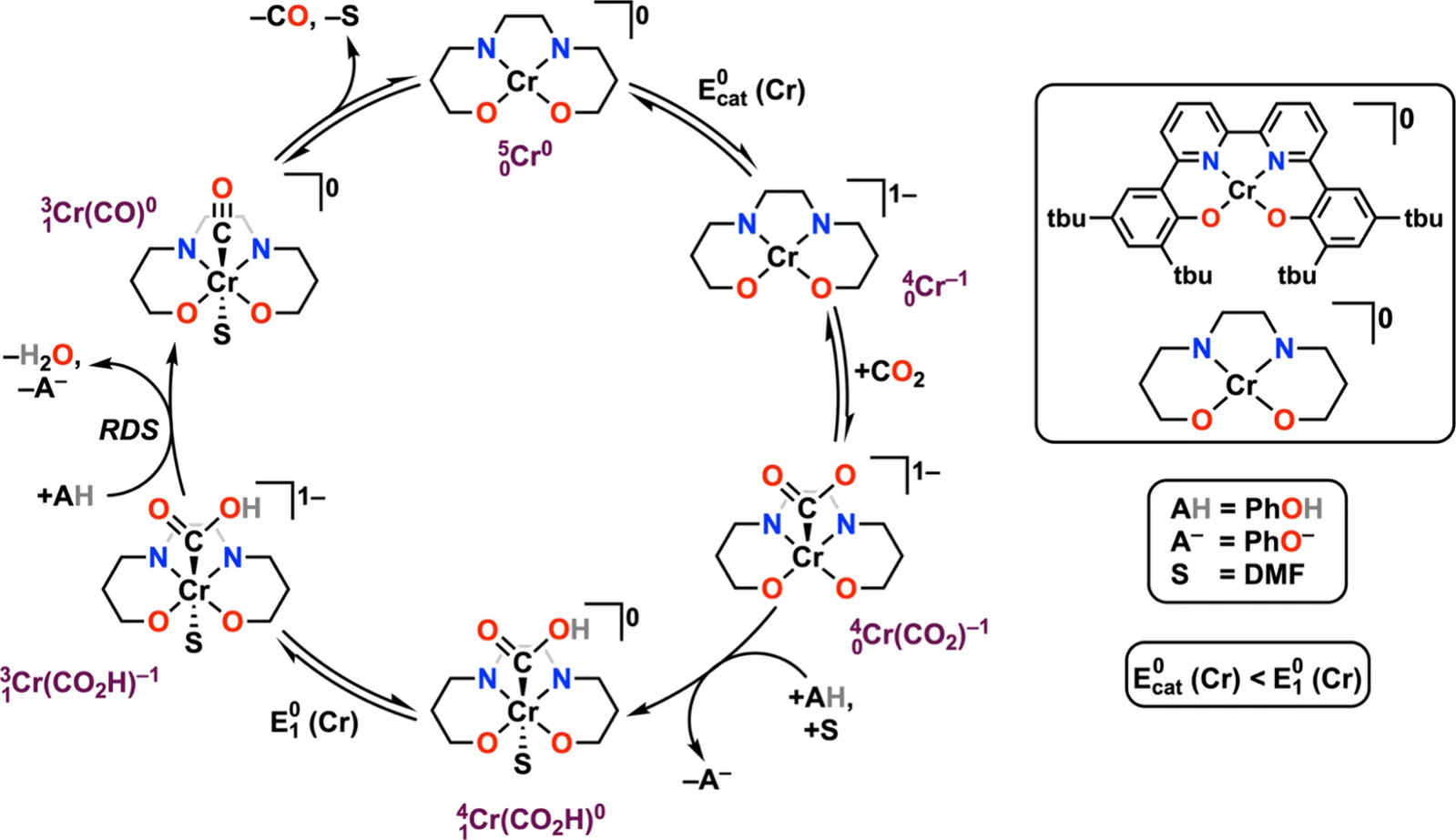
Proposed catalytic mechanism for protic electrocatalytic CO_2_ reduction mediated by Cr­(^*tbu*^dhbpy)­Cl­(H_2_O) (**1**). Adapted with permission from *Accounts of Chemical Research*.^19^ Copyright 2024 American Chemical Society.

To increase catalytic activity of these Cr-based systems for the CO_2_RR, ligand modification and the use of redox mediators (RM) were explored.^14,16,17,20,23,24^ Optimization of the redox-active bipyridine ligand has led to only slight changes in catalytic activity. However, impressive increases in catalytic activity occur with the introduction of a RM. RMs can participate co-catalytically during electrochemical reactions by transferring electrons to a catalytic active site, thus eliminating potential high-energy intermediates.^23^ Sulfone-based RMs were initially developed, based on dibenzothiophene-5,5-dioxide (DBTD).^20^ The addition of DBTD to a CO_2_-saturated electrolyte solution with **1** and PhOH as a proton donor in *N*,*N*-dimethylformamide (DMF) solution resulted in a large increase in current density at potentials corresponding to the reduction of the RM by cyclic voltammetry (CV). Additionally, co-catalysis yielded a higher TOF of 65.3 s^–1^, a 9-fold increase in comparison to the intrinsic catalytic performance of the Cr complex.^20^ The co-catalytic cycle (Figure 2) was proposed to be an inner-sphere mechanism under protic conditions.^14,16,20^ As before, the neutral four-coordinate catalyst is reduced to the monoanionic species (*i*), CO_2_ binds to form [Cr-CO_2_]^−^ (*ii*), protonation occurs and then the species is reduced to form the monoanionic [Cr-CO_2_H]^−^ (*iii*). This is where the co-catalytic cycle differs from the intrinsic one: the one-electron reduced [RM]^−^ can bind to the anionic Cr complex to form species *iv*. This intermediate is stabilized by pancake bonding between the bpy backbone and RM. A second proton transfer allows for the release of the neutral RM and water leaving a monoanionic five-coordinate [Cr-CO]^−^ species (*v*) which quickly releases CO to form the monoanionic four-coordinate Cr species (*i*) and complete the co-catalytic cycle.

**Figure 2:**
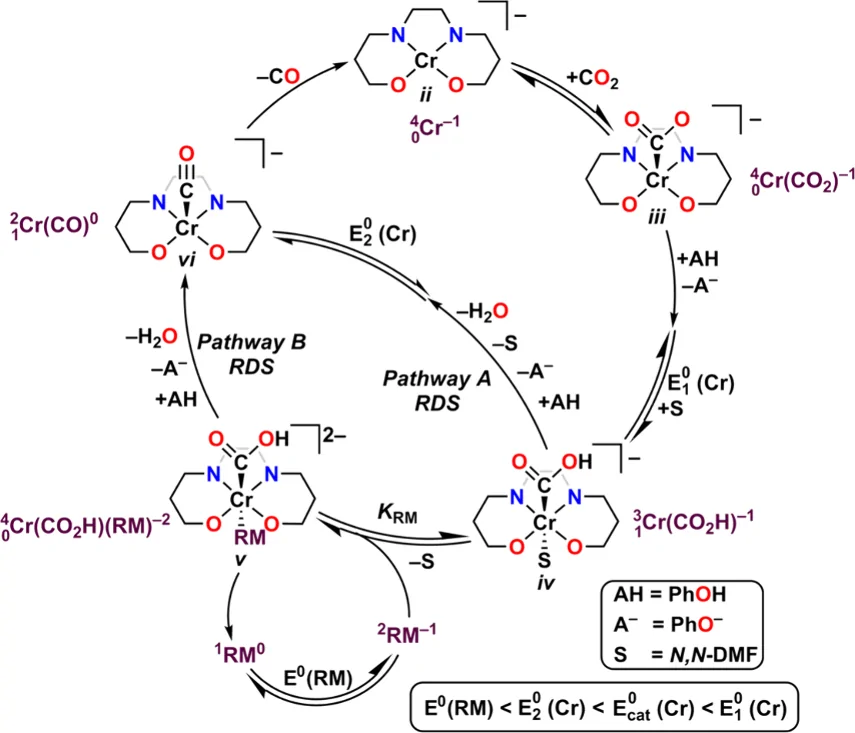
Proposed catalytic mechanism for protic electrocatalytic CO_2_ reduction by Cr (**1**) and protic co-electrocatalytic reduction of CO_2_ by Cr and RM where Cr (**1**) and RM is DBTD or BNTD. Adapted with permission from *Accounts of Chemical Research*.^19^ Copyright 2024 American Chemical Society.

Since the initial studies including RMs, catalyst and RM tuning has been utilized to improve performance and better understand the underlying reactivity. Recent studies have shown that TOF can be maximized when the reduction potential (*E*_1/2_) of the RM is equal to potential where catalysis occurs (*E*_cat/2_) for the catalyst.^17^ For this reason, this paper focuses on work using benzonaphthothiophene-7,7-dioxide (BNTD) since it has a more positive *E*_1/2_ = −1.96 V vs. Fc^+^/Fc than DBTD (*E*_1/2_ = −2.25 V vs. Fc^+^/Fc) (Figure 3).^17^ Although a handful of Cr-based catalysts for CO_2_RR have been synthesized and tested under conditions with and without RMs, this study will focus on **1** for simplicity.

**Figure 3:**
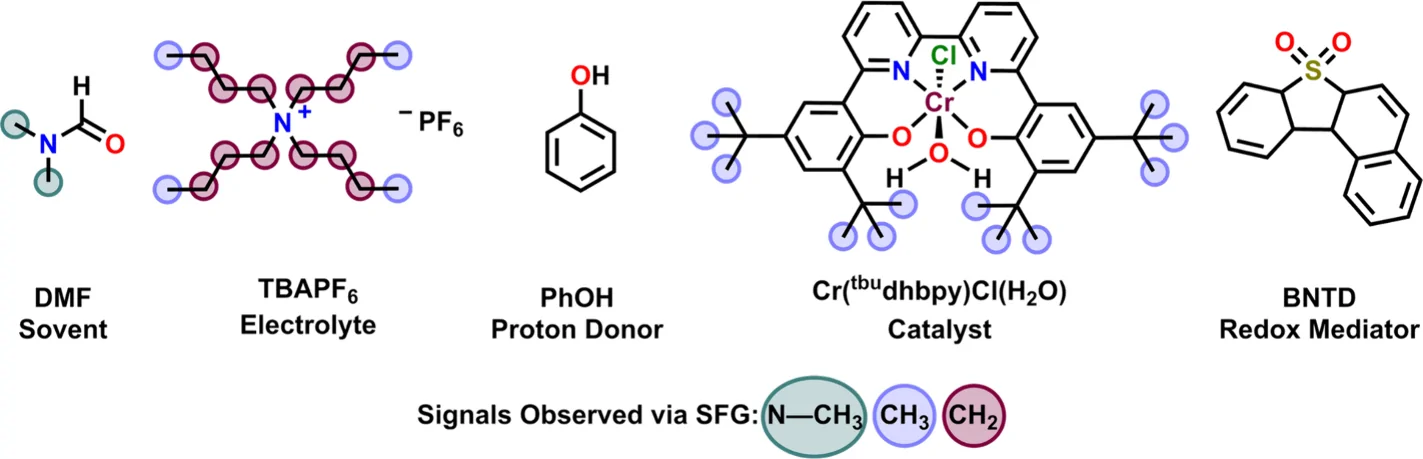
Structures of species under investigation with primary groups contributing to the SFG signals highlighted.

Mechanistic and computational studies have sought to clarify the co-catalytic mechanism between catalyst and RM; however, the direct observation of bimolecular interactions between the RM and metal complex—specifically whether aromatic stacking interactions occur and coordinate covalent bonds form, if these events take place at the interface or within the double layer, and how solvation mediates these events—has been difficult to probe experimentally.^14^ Because these reactions take place at/near electrode interfaces, understanding how molecules arrange and mediate these interactions at applied potentials is foundational to a mechanistic understanding. Here, *surface-specific* spectro-electrochemical approaches are needed to identify potential intermediates and solvation shells as they are formed and evolve *in situ*. In this work, Sum Frequency Generation (SFG) vibrational spectroscopy is used to report on surface population, solvation, and potential dependent reorganization that underly the catalytic chemical conversion discussed above. Coupling this surface specificity of SFG with *in situ* electrochemical measurements provides a glimpse into the competitive role of solvation in electrochemical transformations that is necessary to advance energy efficient transformations.^25–28^

In order to probe the electrochemical behavior of **1** with and without BNTD as a RM while investigating the surface chemistry at the electrode using SFG, a flow cell was manufactured using PEEK, where the working electrode was a 20 nm Au thin film sputtered atop a quartz disk with a 5 nm Ti adhesion layer, as described in the Supplementary Information. The counter and pseudo-reference electrodes used in all experiments were a polished stainless steel disk and Ag/AgCl painted on copper tape, respectively. This approach differs from previous reports that direct the incident laser pulses through the liquid instead of favoring a through-electrode geometry.

The electrochemical response of **1** was measured in DMF with 0.1 M tetrabutylammonium hexafluorophosphate (TBAPF_6_) as the supporting electrolyte, similar to previous studies.^12^ Under blank conditions (0.1 M TBAPF_6_/DMF) with either N_2_ or CO_2_ saturation conditions, there are no observed redox events (Figure 4a). With the addition of **1**, under N_2_ saturation, we observe two irreversible features at *E*_p_ = −0.87 and −1.13 V vs Ag/AgCl, respectively, and a quasi-reversible feature at *E*_p_ = −1.36 V vs AgCl (Figure 4a). This is similar to the observed behavior previously reported for the system;^17^ however, in a typical 3-electrode solution setup using a glassy carbon disc working electrode, the first and third redox features are reversible (Figure S8). The loss of reversibility in the flow cell is attributed to the small distance (100 μm) between the working and counter electrode. Because of the smaller solution volume and the smaller distance between the working and counter electrode, it is possible that the reduced species is being swept from the electrode to the electric double layer and is oxidized at the counter electrode, faster than the CV timescale can measure, which would result in sigmodal waveforms.^29^ In the presence of CO_2_, instead of an inert atmosphere, the first redox feature does not change but the second shifts slightly (*E*_p_ = −0.87 and −1.05 V vs Ag/AgCl, respectively) (Figure 4a). However, the third redox feature becomes distorted and unclear as there is an increase in current density, but no true redox wave change is observed of the type that would be expected to occur with CO_2_ binding. There is also the absence of typical S-shape character that we would see with catalysis. This broad peak at *E*_*p*_ *= −*1.5 V vs Ag/AgCl is most likely CO_2_ binding with the reduced Cr species (Cr­(II)­(bpy^*•–*^)), but due to the slow mass-transport the peak is distorted (Figure 4a). Based on previous observations of inactivity with glassy carbon electrodes under these conditions, it seems likely that the Au assists with CO_2_ activation.^30,31^ With the addition of PhOH under both N_2_ and CO_2_ saturation conditions (Figure 4b), we see a sharp increase in catalytic current, consistent with the hydrogen evolution reaction (HER) occurring under the former conditions (most likely originating from the electrode surface) and some combination of HER and CO_2_ reduction under the latter, based on the change in current-voltage profile.

**Figure 4:**
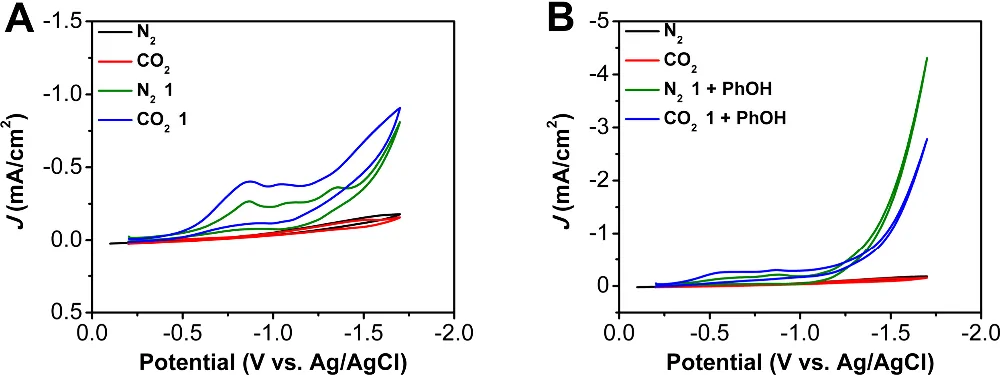
Comparison CVs of Cr­(^*tbu*^dhbpy)­Cl­(H_2_O) **1** under N_2_ and CO_2_ saturation conditions without a proton source (**A**) and under protic conditions (**B**). Conditions: 1.0 mM catalyst, 1.0 M PhOH (when stated), 0.1 M TBAPF_6_/DMF unless otherwise stated; Ti:Au working electrode, stainless-steel counter electrode, Ag/AgCl reference electrode; 50 mV/s scan rate.

Next, the electrochemical system was probed by introducing the redox mediator, BNTD. In the flow cell, BNTD had an irreversible redox feature at *E*_p_ = −1.61 V vs Ag/AgCl under N_2_ saturation (Figure S7). In the presence of CO_2_, we see an additional irreversible feature at *E*_p_ = −0.92 V vs Ag/AgCl and the more negative feature becomes quasi-reversible at *E*_p_ = −1.58 V vs Ag/AgCl (Figure S7). Much like in previous experiments in a standard cell setup, the *E*_*p*_ of **1** and RM were found to almost overlap.^12,17^ When BNTD was added to the solution with **1** and PhOH, under N_2_ saturation, we see an increase in current density at −1.63 V vs Ag/AgCl, most likely attributed solely to the response of BNTD (Figure 5a). However, under CO_2_ saturation conditions, we see a large increase in current density with S-shaped character consistent with catalysis at an *E*_cat/2_ = −1.60 V (Figure 5b). This peak is well-defined, indicating that even in the small cell volume, a steady-state catalytic response occurs with the RM in solution.

**Figure 5:**
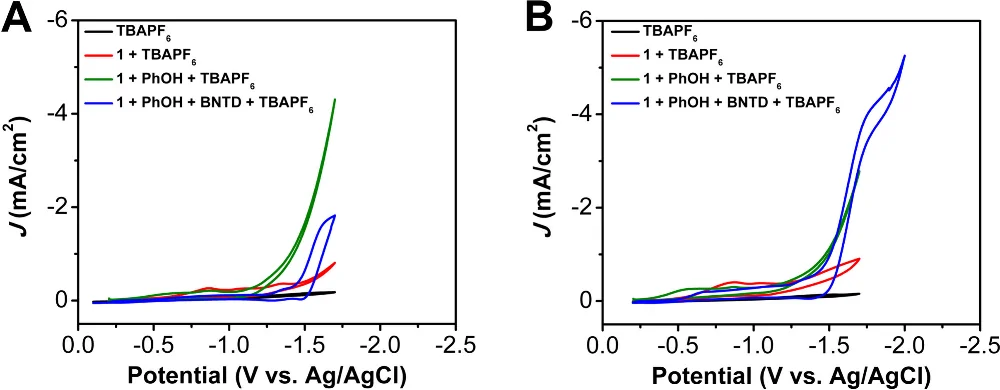
Comparison CVs of Cr­(^*tbu*^dhbpy)­Cl­(H_2_O) **1** under N_2_ (**A**) and CO_2_ (**B**) saturation conditions without a proton source (Red), under protic conditions (Green) and under protic conditions with a RM present (Blue). Conditions: 1.0 mM catalyst (when stated), 1.0 M PhOH (when stated), 2.5 mM BNTD (when stated), and 0.1 M TBAPF_6_/DMF; Ti:Au working electrode, stainless-steel counter electrode, Ag/AgCl reference electrode; 50 mV/s scan rate.

To probe the chemical species at the interfaces in the various electrochemical scenarios above, we used SFG measurements at OCV and −1.7 V vs. Ag/AgCl to probe field induced changes in speciation and solvation at a resting state and during active CO_2_ reduction conditions. When the incident infrared light is resonant with vibrational transitions in the surface localized molecules, there is an enhancement in the SFG response, thereby mapping a chemical spectrum akin to IR or Raman spectroscopy, but only reporting on molecules at interfaces where symmetry is broken. Details of the SFG measurements are given in the Supporting Information. It should be noted that the applied fields can alter the observed line shapes of the measured vibrational modes by way of interference with the nonresonant contribution of Au (which may be potential dependent) and contribution from field-polarized bulk solvent via the so-called *χ*_*bulk*_^(3)^-effect.^32^ To address potential changes to these background contributions, all data were fit to eq S2 (see Supporting Information), where we previously showed how a simplified fitting function can be defined that includes net contributions from complex, time-evolving background responses.^33^ All discussions of peak positions and amplitudes are described via this analytical context.

Given the electrochemical reaction in Figure 2 is chemically complex, the catalytic system is systematically simplified to identify both chemical and potential-driven changes at the electrode interface. The focus of this work is to identify changes in solvation mediating catalytic behavior, so measurements were made in the CH stretching region as to probe terminal methyl groups on the DMF solvent and their response to local fields and interfacial species. DMF was used as the solvent as the CO_2_RR has been validated in this solvent system previously^12^ and the CH stretches associated with DMF (2903 cm^–1^ and 2940 cm^–1^) and the electrolyte, TBAPF_6_ (CH_2_–*ss* at 2850 cm^–1^ and CH_3_-*ss* at 2875 cm^–1^), are sufficiently different that the chemical origins can be readily resolved. Here, we present measurements of TBAPF_6_/DMF solutions at the Au electrode interface at OCV (Figure 6A, blue) to assign baseline vibrational resonances arising from the solvent and supporting electrolytes. The features observed at ∼2903 cm^–1^ and ∼2940 cm^–1^ are assigned to methyl ((CH_3_)_2_-N) symmetric stretches of DMF in the *trans* and *cis* conformations, respectively. This assignment agrees with previous reports probing air/DMF interfaces^34^ and is supported by control measurements at quartz/neat DMF interfaces provided in Figure S1 and S2. On application of a −1.7 V vs Ag/AgCl potential, the SFG spectrum changes such that only the signal from the *cis* conformer is observed, though with a notably diminished overall intensity. The change in conformational preference and intensity is reflective of field-driven solvent rearrangement, a key step in subsequent electron transport aligning the methyl groups more parallel to the interfacial plane, sketched in Figure 7, resulting in azimuthal averaging a diminished SFG response.

**Figure 6:**
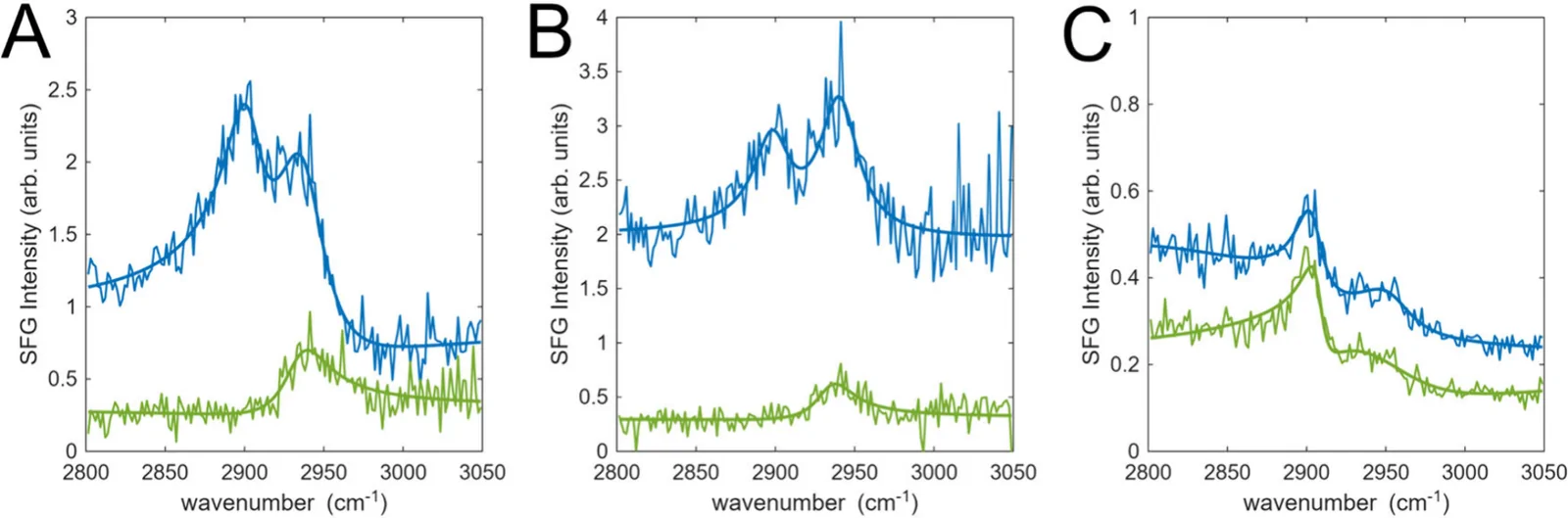
SFG spectra at OCV (blue) and −1.7 V vs Ag/AgCl (green), under CO_2_ saturation, for various solutions in contact with a Ti:Au electrode. DMF with supporting electrolyte is measured (**A**), to this, the Cr catalyst is added (**B**), and finally, a redox mediator with phenol proton source is measured (**C**). Change in band intensities report on conformational difference taken on by the solvent in response to surface and near surface species and in response to applied potential.

**Figure 7:**
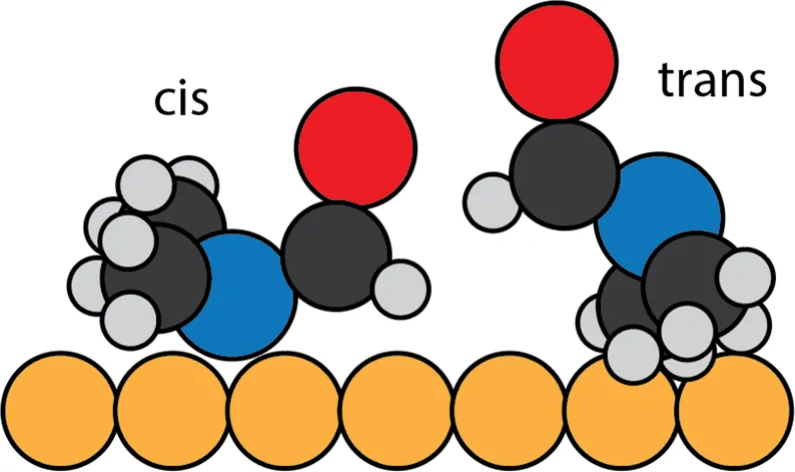
DMF configurations at electrode surface with and without an applied potential.

Perhaps surprisingly, signals from the quaternary ammonium salt are absent in the SFG spectra in Figure 6A, pointing to their structural symmetry and localization in the electric double layer as rendering them SFG inactive. We note that TBA^+^ cations respond differently to charged interfaces in nonaqueous systems compared with aqueous. In aqueous systems, TBA^+^ strongly adsorbs onto electrodes as the hydrophobicity provides additional driving force to tightly pack within the Helmholtz layer and form a hydrophobic interlayer.^35^ However, aprotic polar solvents such as DMF solvate the alkyl chains, inhibiting physisorption. In fact, the strong polar nature of DMF enables it to compensate for surface charge through molecular rearrangement at the interface.^36^ This is supported by application of a −1.7 V vs Ag/AgCl potential, which should drive cation adsorption and increase would be signals from the associated alkyl groups. However, the lack of alkyl signals (∼2850–2880 cm^–1^) measured at negative potential indicates these species remain in a centrosymmetric configuration regardless of applied field. This observation parallels previous SFG work probing TBA^+^ + SO_4_^2–^ air/water interfaces,^37^ where no alkyl stretches were observed, though perturbations to interfacial OH stretching modes from the cation were used to indirectly detect ion localization. In this context, the applied potential drives the conformational preference of DMF to favor the *cis* isomer in accommodating species driven to the electric double layer.

On inclusion of CO_2_ into solution with the Cr catalyst, the SFG response, shown in Figure 6B, changes only subtly by reporting on difference in interfacial solvation by way of different conformational mixtures of *cis*/*trans-*DMF. The difference in DMF peak ratios on inclusion of the catalyst parallels studies that indirectly track molecular interactions by way of perturbing the surface solvation environment, typically via OH stretching vibrations. Here, observations at OCV together with associated changes in conformation with applied potential, suggest that neither the catalyst, nor CO_2_ itself, directly interacts with the electrode surface. Intuitively, the net negative charge taken by the catalyst on CO_2_ uptake should be repelled by the negatively charged surface, indicating again that salient electron transfer pathways are mediated through solvent processes originating from, but not necessarily taking place at, the electrode interface. This conclusion is further supported by the lack of CH_3_ stretches from the *t*-butyl groups on the catalyst, which would appear as strong vibrational bands at ∼2875 cm^–1^. We note here that the detection limits of SFG are sub-monolayer, where trace contaminants, etc., are routinely observed in measurements.^38^ To confirm sensitivity to the electrolytes, experiments were run in non-catalytic conditions in the absence of CO_2_ (Figure S3). Given observation of vibrational markers from the electrolytes in control measurements, the lack of corresponding signals in catalytic systems can be used as a reliable indicator of their exclusion from the surface. The lack of any spectral signature from these otherwise intense vibrations indicates these species are localized away from the surface. This is further supported by the lack of reversible features seen in the CV traces (Figure 4).

These conclusions are additionally supported by inclusion of the redox mediator, BNTD, with the phenol proton source. The SFG spectra in Figure 6C shows that interfacial DMF species adapt to strongly favor the *trans* conformers irrespective of applied potential, as evidenced by the peak at ∼2900 cm^–1^. This is further supported by the fact that PhOH is known to be a H-bonding receptor of the carbonyl;^39,40^ therefore, a trans configuration would be favored such that the carbonyl would face the solution to H-bond with the PhOH presentn. This result suggests that the neutral BNTD is localized near the surface at all potentials and that interfacial DMF adapts to accommodate these species as to facilitate electron transport events away from the surface. Notably, the preference of a *trans* conformer aligns with the notion that electron transfer to BNTD takes place in an outer-sphere manner through the pi-bonding network to form the anion radical, which then coordinates to **1** to conduct an inner-sphere electron transfer.^17,23^ Given the lack of direct adsorption, BNTD and its anion remain unstructured near the surface, as supported by CVs (Figure 5) and by the absence of aromatic stretches at 3000–3100 cm^–1^ given azimuthal in-plane symmetry. On accepting an electron, the redox mediator leaves the interface, as evidenced by control CVs (Figure S7) allowing another species to occupy the surface. Due to the interactions afforded by BNTD, the vibrational resonances are weaker and little change in the SFG spectrum should be observed between OCV and applied potential from the perspective of the solvent—which is indeed observed from SFG measurements (Figure 6C). Together, these results suggest that for common solvents in electrocatalytic reactions, there is a balance needed between space filling and dipolar interactions unless a more surface-active species, e.g., a redox mediator, is available to moderate electron transport events away from the electrode interface.

In summary, through coupled CV and SFG measurements, we determined that DMF dominates the available space at the electrode surface and responds to electrochemical species localized in the double layer by changes in conformational isomers and their orientation when a negative potential is applied. These solvent effects and electron transfer through the solvent are mitigated by the use of an aromatic redox mediator that is able to participate in electron transfer to/from the electrode and the bulkier catalyst at the electrode interface. This was observed in SFG measurements as an indifference to the applied field, yet catalytic enhancement was obvious in the CV measurements. Future work in this field is greatly needed, as there are many unknowns about interactions at the electrode interface during electrochemical experiments, requiring measurements in different spectral regions that are not afforded by quartz supports. Similarly, covalently attaching the catalyst directly to the electrode could enable insight into these electron-transfer events.^25^

## Supplementary Material



## Notes

Description of experimental details, methods, schematic of flow cell, electrochemical data associated with the spectral data in Figure 6, necessary CVs taken in flow cell and spectral data at quartz (PDF)
