# Supplementary material for: Solvation Mediates the Mechanisms of Homogeneous Co-Electrocatalysis at Interfaces
Source: J Phys Chem Lett. 2026 Sep 30;17(41):11940–6. doi: 10.1021/acs.jpclett.6c02610 (PMC13679078; doi:10.1021/acs.jpclett.6c02610)
Supplement: Supplementary file 1 [file jz6c02610_si_001.pdf]

Supporting Information for Publication

Solvation Mediates the Mechanisms of Homogeneous Co-Electrocatalysis at Interfaces

Megan E. Moberg[a], Robert L. Sacci[b,*], Benjamin Doughty[b,*], and Charles W. Machan[a,*]

MEM ORCID 0000-0003-2083-9877; RLS ORCID 0000-0002-0073-5221; BD ORCID 0000-0001-6429-9329; CWM ORCID 0000-0002-5182-1138

[a]*Department of Chemistry, University of Virginia, PO Box 400319, Charlottesville, VA 22904-4319*

[b]*Chemical Sciences Division, Oak Ridge National Laboratory, Oak Ridge, TN 37831*

* - saccirl@ornl.gov; doughtybl@ornl.gov; machan@virginia.edu

This manuscript has been authored by UT–Battelle, LLC, under contract DE–AC05–00OR22725 with the US Department of Energy (DOE). The US government retains and the publisher, by accepting the article for publication, acknowledges that the US government retains a nonexclusive, paid–up, irrevocable, worldwide license to publish or reproduce the published form of this manuscript, or allow others to do so, for US government purposes. DOE will provide public access to these results of federally sponsored research in accordance with the DOE Public Access Plan (http://energy.gov/downloads/doe–public–access–plan).

Materials and Methods

General

All chemicals and solvents (ACS or HPLC grade) were commercially available and used as received unless otherwise indicated. For all air-sensitive reactions and electrochemical experiments, HPLC-grade solvents were used. Gas cylinders were obtained from Praxair (N_2 as 5.0; CO_2 as 4.0).

Electrochemistry

All electrochemical experiments were performed using a BioLogic SP-50 potentiostat. Stainless-steel disks were used as counter electrodes. Ag/AgCl pseudoreference electrodes were made by painting copper tape with Ag/AgCl and letting dry overnight. The tape was then attached to hand-punched Viton spacers (0.1 mm-thick) and used in the flow cell. Working electrodes were made from piranha cleaned quartz (25.4mm dia. x 0.15–0.25mm (0.006-0.01in) thick) that were then placed in a D.C. magnetron sputtering system, in order to adhere thin Ti and then Au layers. All electrochemical experiments were performed in a modified flow cell. Electrochemical solutions were sparged with gas prior to use in the flow cell. $TBAPF_6$ was purified by recrystallization from ethanol and dried in a vacuum oven before being stored in a vacuum desiccator. Controlled potential electrolysis (CPE) experiments were performed in the flow cell following a CV experiment. CPE experiments were held at –1.7 V vs Ag/AgCl, unless otherwise specified, for the entirety of the SFG experiment. The CPE was started prior to SFG experiments.

Sample Preparation

Electrochemical solutions were prepared using N,N-dimethylformamide (DMF) as the solvent, tetrabutylammonium hexafluorophosphate ($TBAPF_6$) as the electrolyte, phenol (PhOH) as the proton donor, Cr(^{tbu}dhbpy)Cl(H_2O) (**1**) as the catalyst, and when relevant, benzonaphthothiophene-7,7-dioxide (BNTD) as the RM. Unless stated otherwise the solutions were saturated with CO_2 and in ratios of: 1.0 mM **1**, 1.0 M PhOH, 2.5 mM BNTD and 0.1 M $TBAPFF_6$/DMF was used for all measurements. A flow cell was used and described in detail below.

Electrode Preparation

Au thin films with Ti adhesion layers were deposited via sputtering onto quartz disks. The metal targets were 2 in. diameter discs (Kurt Lesker, 99.999%). During sputtering, the target-to-substrate distance was maintained at 7.5 cm. The vacuum chamber was pumped down to a base pressure of 1×10^{-6} Torr for at least 3 h. The system was then pressurized to 20 mTorr using 20 sccm flow of Ar gas (99.999%, AirGas), and plasma was ignited and sustained using a 20 W DC power source. The deposition rate was checked using a quartz crystal monitor, and the deposition time was set to achieve 5 nm for Ti and 20 nm for Au.

Sum Frequency Generation (SFG) Measurements

A homebuilt instrument, described in detail previously, was used to perform the SFG measurements. Briefly, collinear narrowband near-infrared (NIR) and broadband IR were incident on the surface of the quartz substrates at ~60° relative to the surface normal to reach the buried quartz/solution or electrode/solution interface. The quartz or electrode substrates were placed in a PEEK flow cell for all measurements. SFG signals generated from the interface of interest were then collimated and filtered before polarization selection. Transmitted light was dispersed and detected with a spectrometer and CCD camera.

The flow cell was designed to be able to take simultaneous electrochemical and SFG measurements. The base of the cell includes an inlet and outlet ports for sample flow. A stainless-steel counter electrode with a copper tape attachment is placed, spacers are used to form a well for the solution to pool. The Ag/AgCl reference electrode with a copper tape attachment is used and thin film quartz with Ti:Au layer is used as a working electrode. The lid has a cutout for the SFG laser measurements to reach the electrode/solution interface. A diagram of the setup can be found below in **Schematic S1**.

All measurements were performed at the SSP polarization where the SFG and NIR sources were S-polarized, and the IR source was P-polarized, report predominantly on symmetric stretches more parallel to the surface normal. Spectra were collected for 240 seconds. For SSP measurements, data were collected with the IR centered at 2900 cm^{-1}. The resulting spectra were background subtracted by a gold reference spectra taken at the same center IR frequencies in the flow cell. SFG data were analyzed, per the work of Geiger and coworkers,[1] where the measured SFG intensity (I_{SFG}) is given by:

$$I_{SFG} \propto \left| \sum_q \frac{A_q}{\omega_{IR} - \omega_q + i\Gamma_q} + \chi_{NR}^{(2)} e^{i\gamma} + \left(\frac{\kappa}{\sqrt{\kappa^2 + (\Delta k_z)^2}} \right) \chi^{(3)} \Phi(0)\, e^{i\varphi} \right|^2 \qquad (Eq.S1)$$

where the first term represents resonant contributions to the SFG response. The second term describes the second order non resonant (NR) response (with phase factor, γ). The final term is the $\chi^{(3)}$, which arises from the surface potential that polarizes bulk molecules (almost always discussed in the context of water) to give an SFG response.

Here, A_q, ω_q, and Γ_q are mode specific amplitudes, resonant frequencies, and peak halfwidths, respectively. The inverse Debye length is described by κ. The inverse SFG coherence length is Δk_z and φ is an additional phase factor that takes the form $\varphi = \arctan[(\Delta k_z)/\kappa]$. $\chi_{bulk}^{(3)} e^{i\phi}$ is the third order susceptibility of the bulk solvent and Φ(0) is the surface potential. Per our previous work on the topic,[2] one can reduce the complexity of this system to a single background response that describes the net system:

$$I_{SFG} \propto \left| \sum_q \frac{A_q}{\omega_{IR} - \omega_q + i\Gamma_q} + \chi_s^{(2)} e^{i\phi} \right|^2 \qquad (Eq.S2)$$

which is used to fit all data presented. Here, the $\chi_s^{(2)} e^{i\phi}$ term contains the non-resonant and $\chi_{bulk}^{(3)}$ response, whereas the phase is comprised of the difference in the non-resonant phase and that of the field ordered phase. As such, changes to line shapes are intrinsically included in the fitting.

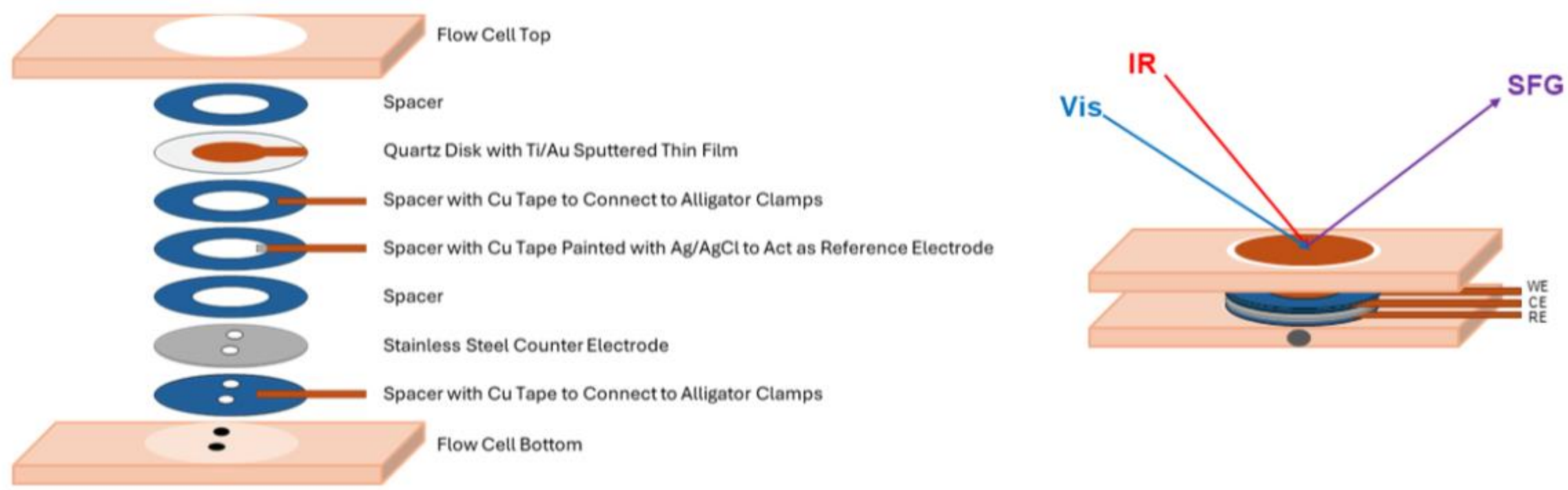

Schematic S1. Illustration of cell setup for *in-situ* electrochemical experiments.

Synthesis and Characterization

Synthesis of 6,6'-di(3,5-di-*tert*-butyl-2-hydroxybenzene)-2,2'-bipyridine, ^{tbu}dhbpy(H)$_2$

The synthesis of ^{tbu}dhbpy(H)$_2$ was carried out as previously reported and the isolated material matched all previous characterization.[3]

Synthesis of Cr(^{tbu}dhbpy)Cl(H_2O) (1)

The synthesis of Cr(^{tbu}dhbpy)Cl(H_2O) was carried out as previously reported and the isolated material matched all previous characterization.[3]

Synthesis of benzonaphthothiophene-7,7-dioxide, BNTD

The synthesis of BNTD was carried out as previously reported and the isolated material matched all previous characterization.[4]

SFG and Electrochemistry

At Quartz

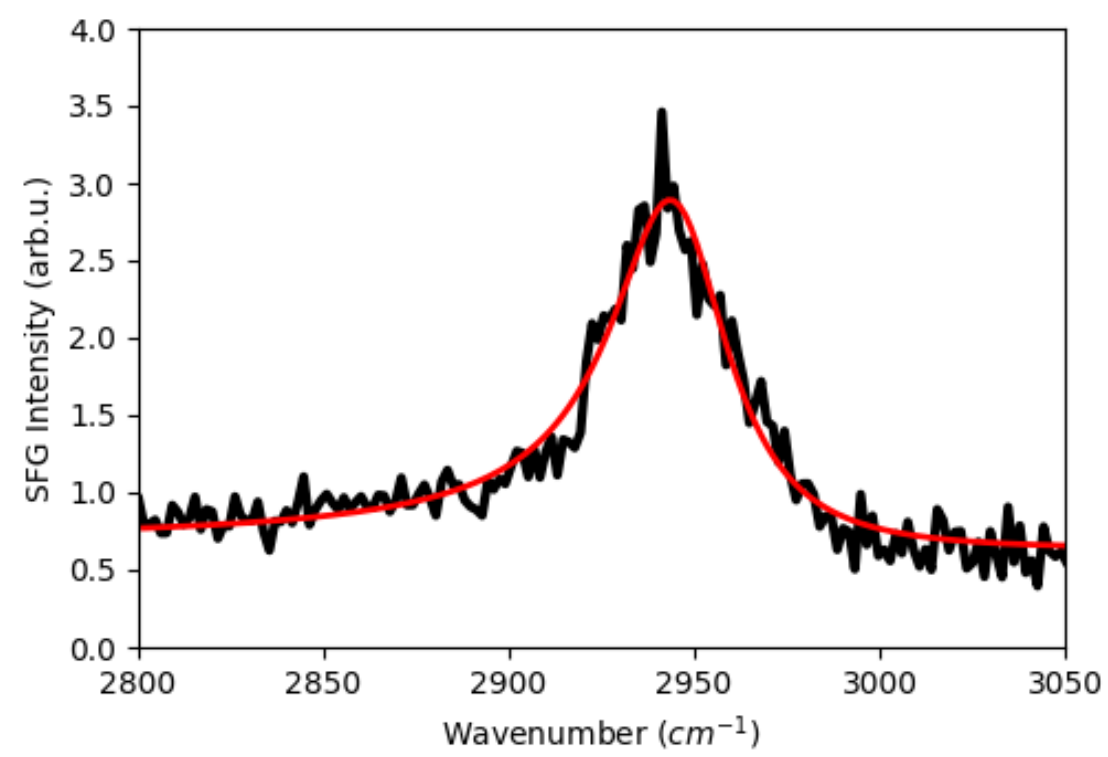

Figure S1. SSP SFG spectra of the quartz/DMF interface under an air atmosphere. Spectra representative of three trials. Black trace corresponds to DMF while the red line overlayed is the fit of the data.

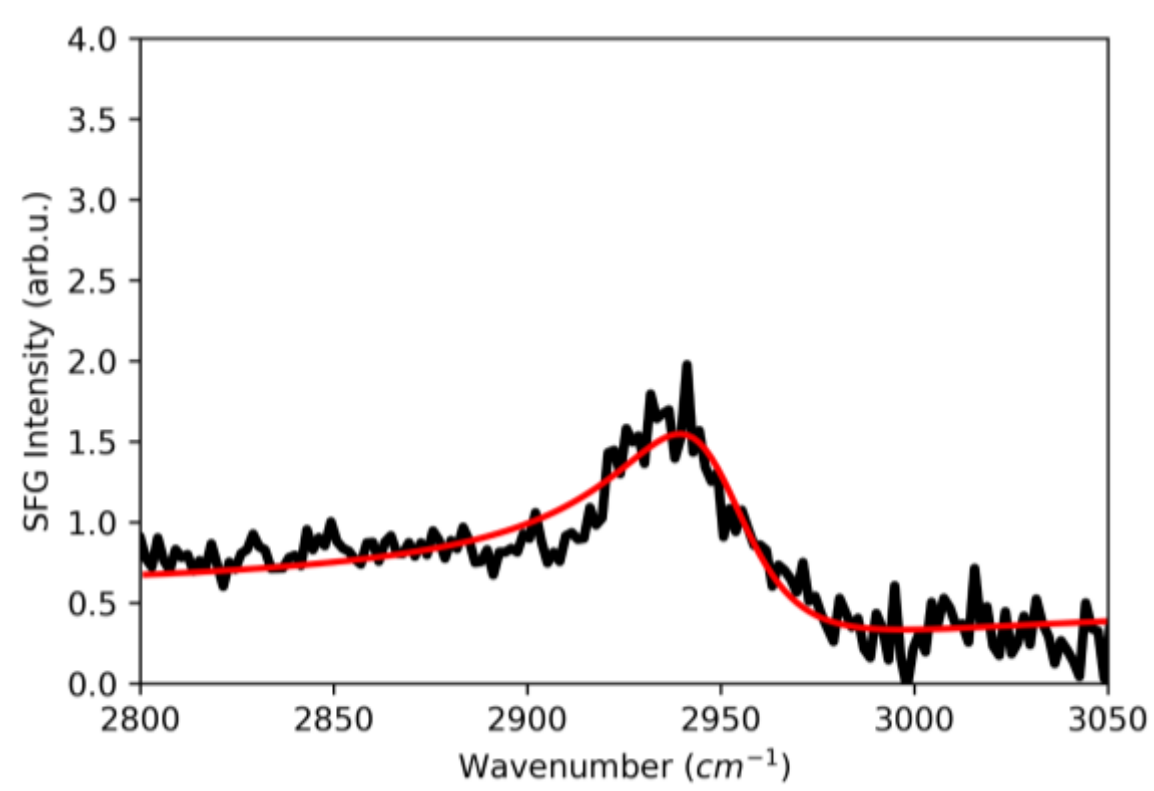

Figure S2. SSP SFG spectra of the quartz/$TBAPF_6$/DMF interface under an air atmosphere. Spectra representative of three trials. Black trace corresponds to $TBAPF_6$/DMF while the red line overlayed is the fit of the data.

At Ti:Au Interface

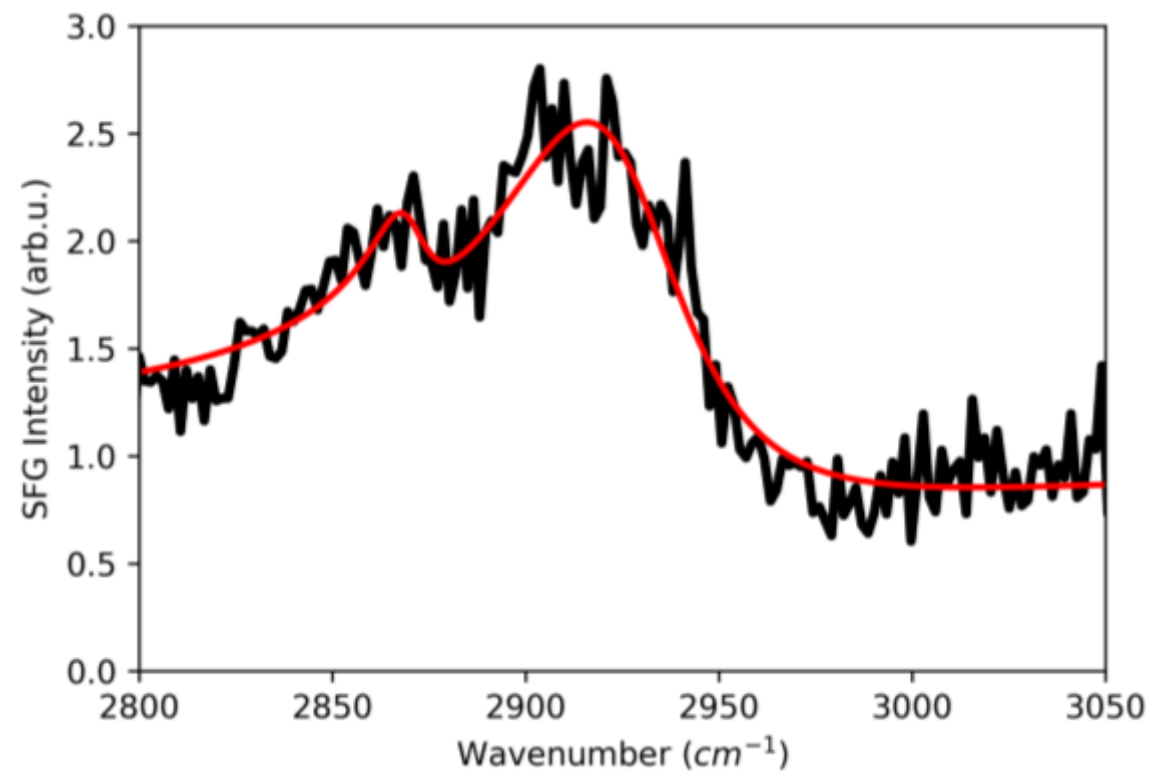

Figure S3. SSP SFG spectra of the Ti:Au $TBAPF_6$/DMF interface at −1.7 V vs Ag/AgCl under N_2 saturation. Spectra representative of three trials. Black trace corresponds to $TBAPF_6$/DMF while the red line overlayed is a fit of the data.

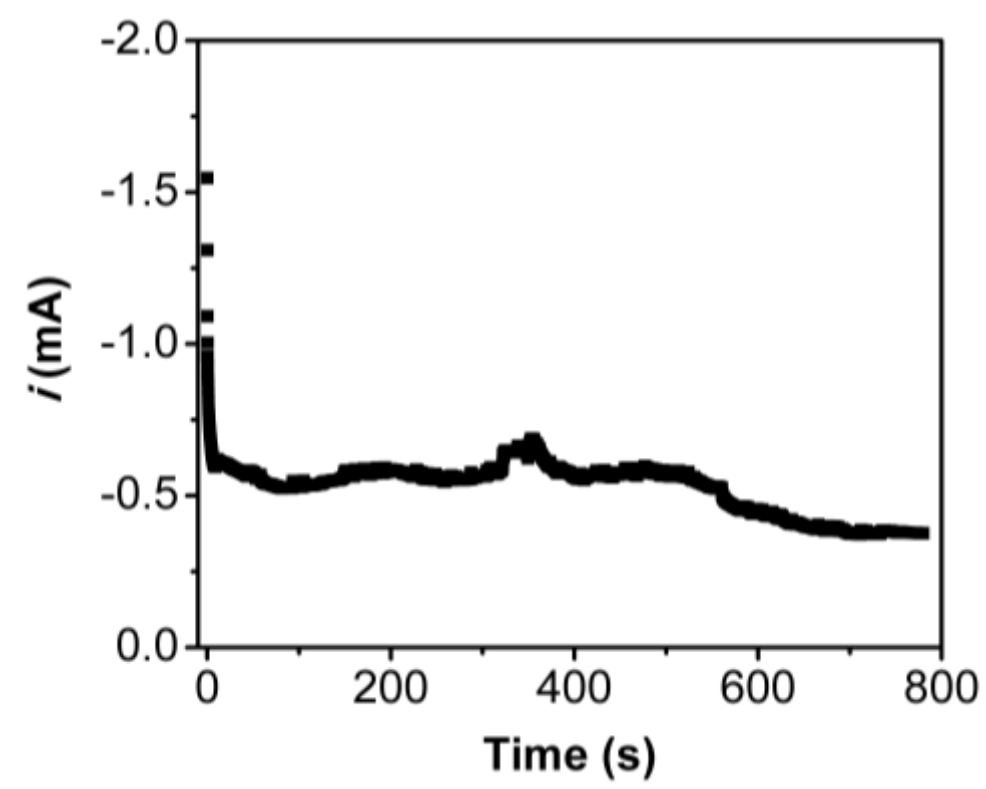

Figure S4. Current versus time trace from CPE experiment for $TBAPF_6$/DMF for the SFG experiment shown in **Figure 6a**. Conditions: 0.1 M $TBAPF_6$/DMF under CO_2 saturation at an applied potential of –1.7 V vs. Ag/AgCl.

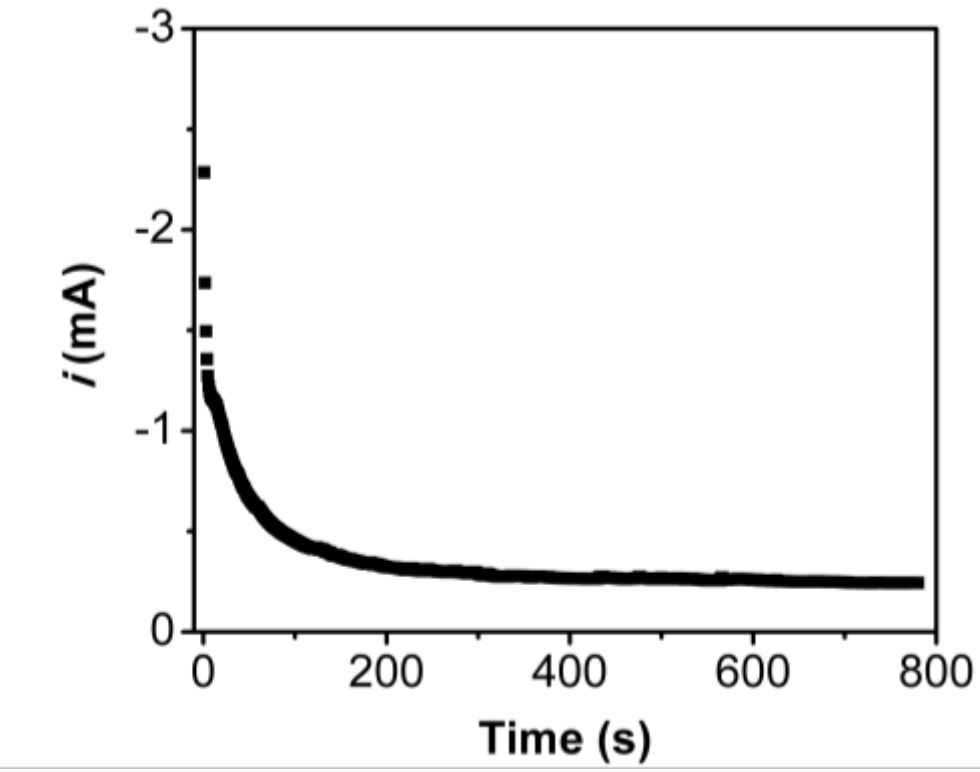

Figure S5. Current versus time trace from CPE experiment for **1** + $TBAPF_6$/DMF for the SFG experiment shown in **Figure 6b**. Conditions: 1.0 mM **1**, 0.1 M $TBAPF_6$/DMF under CO_2 saturation at an applied potential of –1.7 V vs. Ag/AgCl.

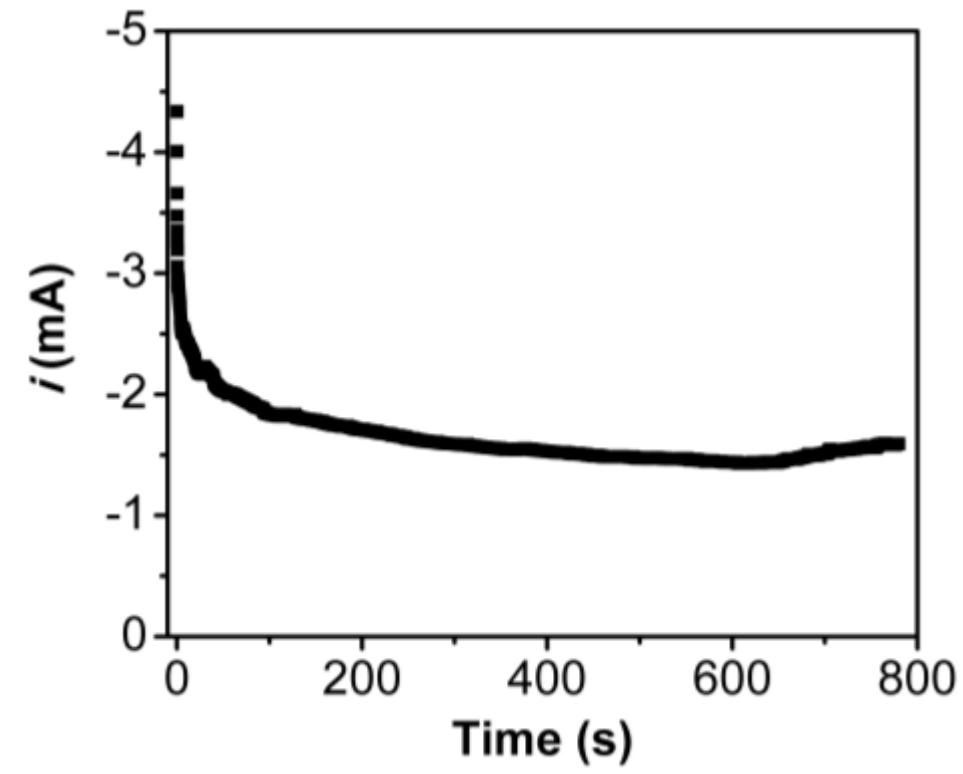

Figure S6. Current versus time trace from CPE experiment for **1** + BNTD + PhOH + $TBAPF_6$/DMF for the SFG experiment shown in **Figure 6c**. Conditions: 1.0 mM **1**, 2.5 mM BNTD, 1.0 M PhOH, 0.1 M $TBAPF_6$/DMF under CO_2 saturation at an applied potential of –1.7 V vs. Ag/AgCl.

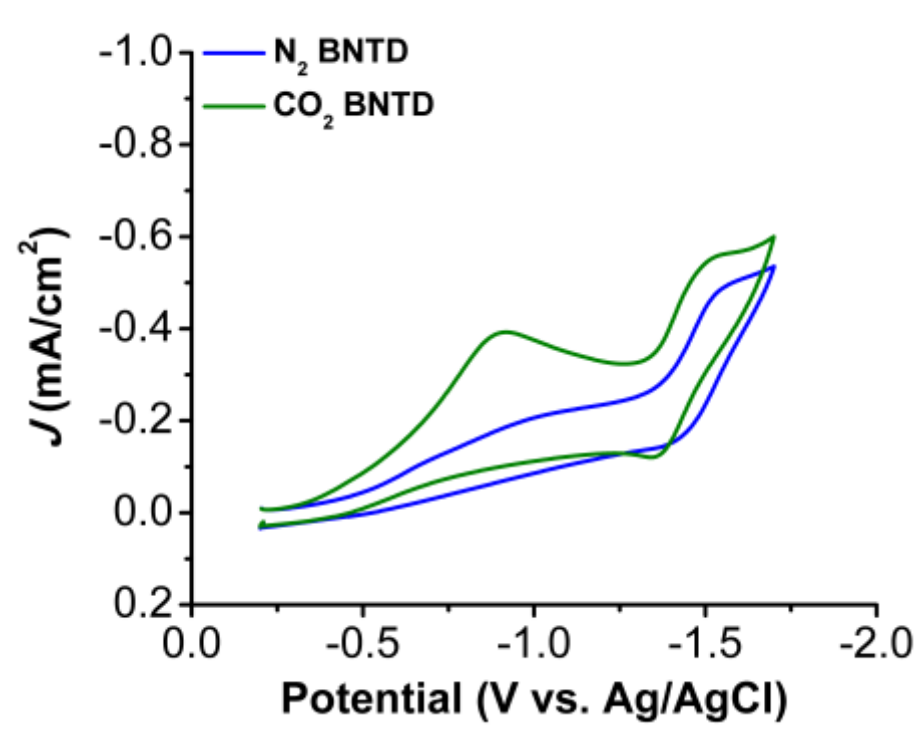

Figure S7. CVs of BNTD under N_2 (Blue) and CO_2 (Green) saturation conditions. Conditions: 2.5 mM BNTD and 0.1 M $TBAPF_6$/DMF; Ti:Au working electrode, stainless-steel counter electrode, Ag/AgCl reference electrode; 50 mV/s scan rate.

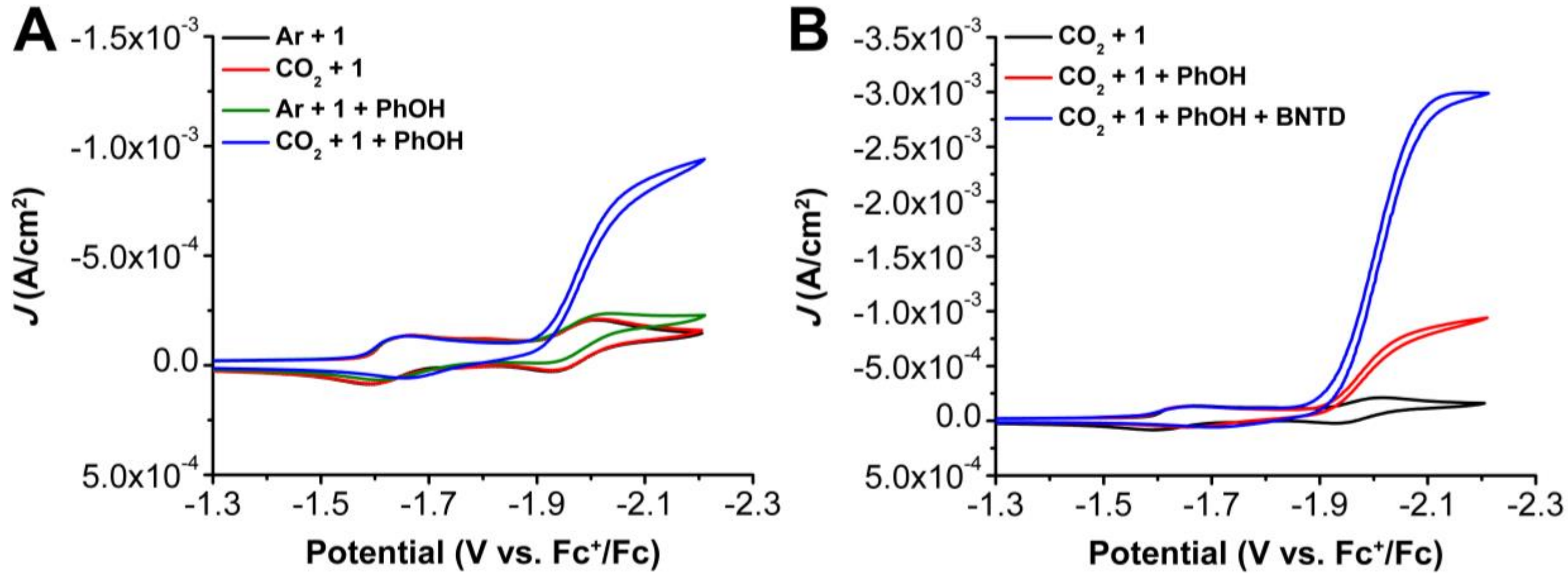

Figure S8. (**A**) Comparison CVs of Cr(^{tbu}dhbpy)Cl(H_2O) (**1**) under Ar and CO_2 saturation conditions with and without 1.0 M PhOH. (**B**) Comparison CVs of Cr(^{tbu}dhbpy)Cl(H_2O) (**1**) under CO_2 saturation conditions with 1.0 M PhOH and 2.5 mM BNTD (when stated). Conditions: 1.0 mM **1**, 0.1 M $TBAPF_6$/DMF; glassy carbon disc working electrode, glassy carbon rod counter electrode, Ag/AgCl pseudoreference electrode, referenced to a Fc^+/Fc internal standard; 100 mV/s scan rate.

References

1. Ohno, P. E.; Wang, H.-f.; Geiger, F. M. Second-order spectral lineshapes from charged interfaces. *Nat. Commun.* **2017**, *8* (1), 1032. DOI: 10.1038/s41467-017-01088-0.
2. Premadasa, U. I.; Bocharova, V.; Lin, L.; Genix, A.-C.; Heller, W. T.; Sacci, R. L.; Ma, Y.-Z.; Thiele, N. A.; Doughty, B. Tracking Molecular Transport Across Oil/Aqueous Interfaces: Insight into “Antagonistic” Binding in Solvent Extraction. *J. Phys. Chem. B* **2023**, *127* (21), 4886-4895. DOI: 10.1021/acs.jpcb.3c00386.
3. Hooe, S. L.; Dressel, J. M.; Dickie, D. A.; Machan, C. W. Highly Efficient Electrocatalytic Reduction of CO_2 to CO by a Molecular Chromium Complex. *ACS Catal.* **2020**, *10* (2), 1146-1151. DOI: 10.1021/acscatal.9b04687.
4. Reid, A. G.; Zelenke, E. A.; Moberg, M. E.; Dickie, D. A.; Machan, C. W. Improving co-electrocatalytic carbon dioxide reduction by optimizing the relative potentials of the redox mediator and catalyst. *Chem. Commun.* **2024**, *60* (63), 8208-8211, 10.1039/D4CC01988A. DOI: 10.1039/D4CC01988A.
